# Vaccines for older adults; the low-hanging fruit of disease prevention: Manuela Wennekes

**DOI:** 10.1093/eurpub/ckac131.407

**Published:** 2022-10-25

**Authors:** MD Wennekes, R Eilers, A Caputo, A Gagneux-Brunon, R Gavioli, F Nicoli, MMM Quatrehomme, Z Vokó, A Timen

**Affiliations:** 1 National Coordination Centre for Communicable Disease Control, RIVM, Bilthoven, Netherlands; 2 Athena Institute, Free University, Amsterdam, Netherlands; 3 Department of Chemical, Pharmaceutical and Agricultural Sciences, University of Ferrara, Ferrara, Italy; 4 Groupe sur l'Immunité des Muqueuses et Agents Pathogènes, University Jean Monnet, Saint-Etienne, France; 5 Department of Infectious Diseases, University Hospital of Saint-Etienne, Saint-Etienne, France; 6 Syreon Research Institute, Budapest, Hungary; 7 Center for Health Technology Assessment, Semmelweis University, Budapest, Hungary; 8 Department of Primary and Community Care, Radboud University Medical Center, Nijmegen, Netherlands; 9 WP4, VITAL, Netherlands

## Abstract

**Background:**

The COVID-19 pandemic highlighted the significance of vaccination for older adults (OA), however, more health benefits could be gained with vaccination against influenza, pneumococcal disease, herpes zoster and tetanus as their uptake remains rather low. As healthcare professionals (HCP) play an important role in the vaccination decision making of OA, this study identifies obstacles in vaccination communication between HCP and OA.

**Methods:**

80 in-depth structured interviews have been conducted with HCPs in Hungary (HU), Italy (IT), the Netherlands (NL) and France (FR). Participants were general practitioners, medical specialists, public health physicians, occupational physicians, pharmacists, geriatricians, specialists elderly care and nurses. The interview included questions on HCPs’ perceptions regarding information provision to OA on vaccines. Data were analyzed cross-country, using thematic analysis.

**Results:**

Preliminary results reveal that a factor hindering HCPs to initiate conversations with OA on vaccines was lack of time (FR, IT, HU, NL). In hospitals this was often due to (acute) clinical problems taking precedence over discussing vaccines (IT, NL). In ambulatory settings the high number of patients waiting to be seen prevented discussing vaccines with OA (HU). Moreover, HCPs sometimes forgot to discuss vaccines with OA (NL, HU, IT). Patient factors hindering the conversation of HCPs on OA vaccines were a negative attitude (IT, HU) and lack of understanding the information provided (IT, HU). Also, misinformation on vaccines (FR, HU), as well as anti-vax beliefs from patients (NL) or their relatives (FR, IT) hampered the conversation on vaccines. HCPs mentioned their need to learn communication skills to convince OA on vaccines (FR, IT, HU).

**Conclusions:**

HCPs encounter various obstacles in communicating with OA about vaccines. Lack of time and not recognizing the opportunity to discuss vaccines are important barriers for initiating vaccine conversations.

**Key messages:**

• Providing HCPs with communication strategies is important to support HCPs in discussing vaccines with OA.

• Reminder systems are important to help HCPs remember address vaccination.

